# Artificial intelligence in clinical and genomic diagnostics

**DOI:** 10.1186/s13073-019-0689-8

**Published:** 2019-11-19

**Authors:** Raquel Dias, Ali Torkamani

**Affiliations:** 1 The Scripps Translational Science Institute, The Scripps Research Institute, 3344 North Torrey Pines Court Suite 300, La Jolla, CA 92037 USA; 2 Department of Integrative Structural and Computational Biology, The Scripps Research Institute, 3344 North Torrey Pines Court Suite 300, La Jolla, CA 92037 USA

## Abstract

Artificial intelligence (AI) is the development of computer systems that are able to perform tasks that normally require human intelligence. Advances in AI software and hardware, especially deep learning algorithms and the graphics processing units (GPUs) that power their training, have led to a recent and rapidly increasing interest in medical AI applications. In clinical diagnostics, AI-based computer vision approaches are poised to revolutionize image-based diagnostics, while other AI subtypes have begun to show similar promise in various diagnostic modalities. In some areas, such as clinical genomics, a specific type of AI algorithm known as deep learning is used to process large and complex genomic datasets. In this review, we first summarize the main classes of problems that AI systems are well suited to solve and describe the clinical diagnostic tasks that benefit from these solutions. Next, we focus on emerging methods for specific tasks in clinical genomics, including variant calling, genome annotation and variant classification, and phenotype-to-genotype correspondence. Finally, we end with a discussion on the future potential of AI in individualized medicine applications, especially for risk prediction in common complex diseases, and the challenges, limitations, and biases that must be carefully addressed for the successful deployment of AI in medical applications, particularly those utilizing human genetics and genomics data.

## Background

Artificial intelligence (AI) is the simulation of intelligence in a non-living agent. In the context of clinical diagnostics, we define AI as any computer system that can correctly interpret health data, especially in its native form as observed by humans. Often, these clinical applications adopt AI frameworks to enable the efficient interpretation of large complex datasets. These AI systems are trained on external health data that have usually been interpreted by humans and that have been minimally processed before exposure to the AI system, for example, clinical images that have been labeled and interpreted by a human expert. The AI system then learns to execute the interpretation task on new health data of the same type, which in clinical diagnostics is often the identification or forecasting of a disease state.

AI interpretation tasks can be grouped into problem classes such as computer vision, time series analysis, speech recognition, and natural language processing. Each of these problems is well suited to address specific types of clinical diagnostic tasks [1]. For example, computer vision is useful for the interpretation of radiological images, time series analysis is useful for the analysis of continuously streaming health data such as those provided by an electrocardiogram [2], speech-recognition techniques can be used for detection of neurological disorders [3], and AI-based natural language processing can be helpful in the extraction of meaningful information from electronic health record (EHR) data [4]. In some areas, the association between problem classes and diagnostic tasks may not be as obvious; for example, techniques from computer vision are also useful for the identification of functional regulatory elements in the human genome, where they can be used to identify recurrent motifs in DNA sequences in a manner analogous to that in which pixel patterns are detected in images by convolutional neural networks (CNNs; described in the next section) [5].

Many of these problems have been addressed by a specific group of AI algorithms known as deep learning, which can learn interpretable features from large and complex datasets by using deep neural network architectures. Neural networks are computational systems of artificial neurons (also called ‘nodes’) that transmit signals to one another, often in interconnected layers. The layers that are not the input or output layer are termed the ‘hidden’ layers. A deep neural network consists of many hidden layers of artificial neurons. Neural networks often take as input the fundamental unit of data that it is trained to interpret: for example, pixel intensity in images; diagnostic, prescription, and procedure codes in EHR data; or nucleotide sequence data in genomic applications [6]. In other words, unlike most machine-learning approaches, minimal or no human extraction and definition of predictive features are required. A multitude of these simple features are combined in successive layers of the neural network in a variety of ways, as designed by the human neural network architect, in order to represent more sophisticated concepts or features of the input health data. Ultimately, the output of the neural network is the interpretation task that the network has been trained to execute. For example, successive layers of a computer vision algorithm might learn to detect edges in an image, then patterns of edges that represent shapes, then collections of shapes that represent certain objects, and so on. Thus, AI systems synthesize simple features into more complex concepts to derive conclusions about health data in a manner that is analogous to human interpretation, although the complex concepts used by the AI systems are not necessarily recognizable or obvious concepts to humans.

In this review, we describe the recent successes and potential future applications of AI, especially deep learning, in clinical diagnostics, with a focus on clinical genomics. We provide a brief overview of AI algorithms and the classes of problems that they are well suited to address. Next, we provide a more detailed review of how AI has been used to accomplish a variety of clinical genomics tasks, including variant calling and annotation, variant impact prediction, and phenotype-to-genotype mapping. Finally, we end by discussing the potential future applications and challenges of AI in genotype-to-phenotype prediction, especially as it relates to common complex diseases and individualized medicine.

## Artificial intelligence and its applications

The AI algorithms deployed today for clinical diagnostics are termed ‘narrow’ or ‘weak’ AI. These AI algorithms are trained to perform a single task: for example, to classify images of skin lesions into diagnostic categories or to provide a molecular diagnosis from a combination of genomic and phenotypic data. These algorithms do not display general intelligence and are not flexible enough to address other clinical diagnostic tasks. However, transfer learning approaches can be used to adapt a fully trained AI algorithm to accomplish closely related tasks. This is best exemplified by image-based diagnostic AI algorithms that benefit from advances in computer vision and neural networks trained for general image recognition tasks. Thus, the first step in the design of clinical diagnostic AI algorithms usually involves mapping the specific diagnostic task to a more general problem class. Here, we review these problem classes and briefly highlight the intersection of these techniques with genomics.

### Computer vision

**Fig. 1:**
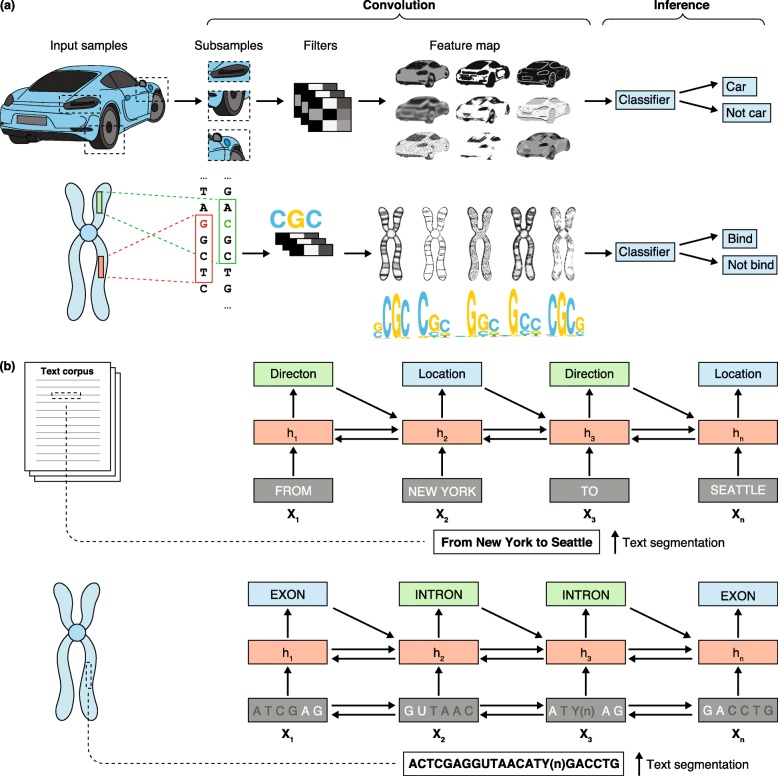
Examples of different neural network architectures, their typical workflow, and applications in genomics. **a** Convolutional neural networks break the input image (*top*) or DNA sequence (*bottom*) into subsamples, apply filters or masks to the subsample data, and multiply each feature value by a set of weights. The product then reveals features or patterns (such as conserved motifs) that can be mapped back to the original image. These feature maps can be used to train a classifier (using a feedforward neural network or logistic regression) to predict a given label (for example, whether the conserved motif is a binding target). Masking or filtering out certain base pairs and keeping others in each permutation allows the identification of those elements or motifs that are more important for classifying the sequence correctly. **b** Recurrent neural networks (RNNs) in natural language processing tasks receive a segmented text (*top*) or segmented DNA sequence (*bottom*) and identify connections between input units (*x*) through interconnected hidden states (*h*). Often the hidden states are encoded by unidirectional hidden recurrent nodes that read the input sequence and pass hidden state information in the forward direction only. In this example, we depict a bidirectional RNN that reads the input sequence and passes hidden state information in both forward and backward directions. The context of each input unit is inferred on the basis of its hidden state, which is informed by the hidden state of neighboring input units, and the predicted context labels of the neighboring input units (for example, location versus direction or intron versus exon)

Computer vision is an interdisciplinary field that focuses on acquiring, processing, and analyzing images and/or video. Computer vision algorithms ingest high-dimensional image data and synthesize (or ‘convolute’) it to produce numerical or symbolic representations of concepts that are embedded in the image. This process is thought to mimic the way humans identify patterns and extract meaningful features from images. The main steps in computer vision consist of image acquisition, pre-processing, feature extraction, image pattern detection or segmentation, and classification. Deep-learning algorithms such as CNNs have been designed to perform computer vision tasks. In simplified terms, a typical CNN tiles an input image with small matrices known as kernel nodes or filters. Each filter encodes a pixel intensity pattern that it ‘detects’ as it convolves across the input image. A multitude of filters encoding different pixel intensity patterns convolve across the image to produce two-dimensional activation maps of each filter. The pattern of features detected across the image by these filters may then be used to successively detect the presence of more complex features (Fig. 1).

Surveillance, image recognition, and autonomous vehicles are some of the major applications of computer vision. In clinical diagnostics, the first applications of AI in healthcare to be cleared by the US Food and Drug Administration (FDA) have been dominated by applications of computer vision to medical scans (for example, magnetic resonance imaging (MRI) or positron emission tomography images), and pathology images (for example, histopathological slides). The first medical imaging applications include the automated quantification of blood flow through the heart via cardiac MRI [7], the determination of ejection fraction from echocardiograms [8], the detection and volumetric quantification of lung nodules from radiographs [7], the detection and quantification of breast densities via mammography [9], the detection of stroke, brain bleeds, and other conditions from computerized axial tomography [10, 11], and automated screening for diabetic retinopathy from comprehensive dilated eye examination [12, 13]. Imaging applications in pathology include an FDA-cleared system for whole-slide imaging [14], and promising approaches to the automated classification of dermatological conditions [15], as well as numerous other whole-slide imaging and AI systems in development that are expected to dramatically enhance the efficiency of pathologists [16].

Computer vision can also inform clinical genomic testing. For example, deep learning of lung cancer histopathological images is able to identify cancer cells, determine their type, and predict what somatic mutations are present in the tumor [17, 18]. Similarly, facial image recognition can be used to identify rare genetic disorders and to guide molecular diagnoses [19, 20]. Thus, computer vision can extract phenotypic features from medical images in order to provide recommendations for molecular testing in a manner similar to that performed by a skilled pathologist or dysmorphologist. In some cases, AI-based systems have exceeded the capabilities of human experts, for example, by accurately predicting gender from retinal fundus images, a task that human experts would perform no better than random guessing [21].

### Time series analysis

Time series analysis is the processing of temporal data to forecast future observations, to predict the discrete state producing a sequence of observations (for example, normal heart rhythm versus arrythmia), or to detect anomalies within a sequence of observations. More generally, time series analysis can be applied to any ordered data; for example, to DNA sequence that is ordered but not temporally ordered. Time series analysis algorithms ingest data sequences and are generally tasked to learn sequential dependencies. The primary advantage of AI algorithms in time series analysis is an improved ability to detect non-linear and/or multi-step relationships that are not efficiently interrogated by traditional approaches such as hidden Markov models. Deep-learning algorithms, especially recurrent neural networks (RNNs), have been designed for sequence analysis tasks. A typical RNN includes some form of ‘memory’, in which prior inputs in a sequence influence future output. This is achieved by linking the hidden state of an input to the hidden state of the next input (Fig. 1). Extensions of this concept, which are implemented in specialized networks such as long short-term memory networks (LSTMs), add network elements that enhance the ability of the network to ‘remember’ long-term dependencies in the input data. CNNs are often applied to time series data when the task is to define the discrete state, or context, that produces the sequential data pattern.

Time series analysis has major applications in the forecasting of equity prices, weather conditions, geological events, and essentially any future event of interest. In clinical diagnostics, time series AI algorithms can be applied to medical devices producing continuous output signals, with the application of electrocardiograms being an especially active area of interest. AI applied to electrocardiograms can detect and classify arrythmias [22], especially atrial fibrillation [23], as well as cardiac contractile dysfunction [24], and blood chemistries linked to cardiac rhythm abnormalities [25]. When applied to genomic sequence data, AI time series algorithms appear to be especially effective at detecting functional DNA sequence elements that are indicative of gene splicing [26, 27], large-scale regulatory elements [28], and gene function [29].

### Automatic speech recognition

Automatic speech recognition includes a group of methodologies that enable the interpretation of spoken language. Speech-recognition algorithms ingest raw sound waves from human speech and process them to allow the recognition of basic elements of speech including tempo, pitch, timbre, and volume, as well as more complex features of speech including the spoken language, words, and sentences [30]. More advanced speech-recognition algorithms can identify sophisticated features from audiological data, such as mood changes or emotional states [31, 32]. Because of the temporal complexity of speech, traditional speech-recognition algorithms have typically relied on separate models to reassemble meaning from spoken language. These steps include segmenting audio into distinct units of sound (for example, phonemes), connecting those sound units into language units (for example, words), and assembling those language units into more complex language elements (for example, phrases) to extract meaning. Recent advances in AI algorithms that address temporal sequences through sequence-to-sequence attention-based and recurrent neural network transducer-based approaches now allow for these tasks to be executed in a single model with streaming output [33, 34]. In sequence-to-sequence models, for example, a neural network can map the sequences of phonemes produced by an acoustic model into sequences of words, or a sequence of words can be translated into another language. Thus, sequence-to-sequence and other speech-recognition models can also act as powerful tools for the communication of medical and health information across language barriers.

Voice command and virtual assistant systems are the major applications of speech recognition. Speech-recognition algorithms have not yet found widespread use in clinical diagnostics but they have shown great promise in the detection of neurological conditions that are often challenging to diagnose with traditional clinical tools. In these clinical applications, the same general speech-recognition strategies are used, but the outcome targeted by the final classification step is a disease phenotype that is typically associated with characteristics of speech (tone, tempo, pitch, and so on) and not necessarily the content of the language. Speech recognition has been successfully applied to the detection of diseases with an obvious influence on speech, notably chronic pharyngitis [35], and of diseases with a less obvious influence on speech, including Alzheimer’s disease [3], Parkinson’s disease [36], major depressive disorder [37], posttraumatic stress disorder [38], and even coronary artery disease [39]. Like imaging, speech recognition can detect potential genetic disorders and inform downstream clinical testing. In addition, speech recognition can be used as a tool to streamline the use of EHRs through automatic transcription, benefitting clinicians and patients and enabling natural language processing (NLP) analysis [40, 41], as described in the next section.

### Natural language processing

NLP is the computational extraction of meaning from natural human language. These algorithms take as input a document, or potentially the output from automatic speech recognition, and output a useful transformation of the document. This transformation could be language translation, document classification, summarization, or extraction of higher-level concepts described by the text. Typical NLP algorithms involve syntactic analysis, which involves parsing the written text in a variety of ways to extract useful computational representations of language (by sentence breaking, tagging parts of speech, and standardizing inflected word forms, for example), followed by semantic analysis to extract meaning and/or the identification of named entities from the text. A wide variety of neural network architectures have been developed for NLP depending upon the target outcome, from sequence-to-sequence networks and other RNN variants for language translation [42], to CNNs to extract higher-level interpretations of the text [43].

A major challenge that is addressed by NLP is the variety of synonyms, phrases, and interrelated concepts that can be used to express a singular meaning. This problem is especially pronounced in clinical applications where controlled vocabularies are numerous and in constant flux. Thus, NLP has been effectively used to automatically standardize and synthesize these terms to produce predictions of current and future diagnoses and medical events [4, 44]. Similarly, NLP can be used to make health information more accessible by translating educational materials into other languages or by converting medical terms to their lay definitions [45]. AI-based chatbots have already been deployed to augment the capabilities of genetic counselors to meet rising demands on their time generated by the rapidly expanding volume of clinical and direct-to-consumer genetic testing [46]. In addition, NLP approaches to EHR analysis can overcome the high dimensionality, sparseness, incompleteness, biases, and other confounding factors present in EHR data. For example, NLP has been applied to EHRs to predict patient mortality after hospitalization. In this application, EHR data are converted to a series of patient events streamed into an RNN, which was trained to identify patterns of patient characteristics, diagnoses, demography, medications, and other events that are predictive of near-term patient mortality or hospital readmission [4]. Similarly, when combined with other medical data, predictions of disease severity and therapy efficacy can be made [47]. When combined with genomic data, NLP-based methods have been used to predict rare disease diagnoses and to drive phenotype-informed genetic analysis, resulting in automated genetic diagnoses with accuracy similar to that of human experts [48, 49].

## Artificial intelligence in clinical genomics

Mimicking human intelligence is the inspiration for AI algorithms, but AI applications in clinical genomics tend to target tasks that are impractical to perform using human intelligence and error prone when addressed with standard statistical approaches. Many of the techniques described above have been adapted to address the various steps involved in clinical genomic analysis—including variant calling, genome annotation, variant classification, and phenotype-to-genotype correspondence—and perhaps eventually they can also be applied for genotype-to-phenotype predictions. Here, we describe the major classes of problems that have been addressed by AI in clinical genomics.

### Variant calling

The clinical interpretation of genomes is sensitive to the identification of individual genetic variants among the millions populating each genome, necessitating extreme accuracy. Standard variant-calling tools are prone to systematic errors that are associated with the subtleties of sample preparation, sequencing technology, sequence context, and the sometimes unpredictable influence of biology such as somatic mosaicism [50]. A mixture of statistical techniques including hand-crafted features such as strand-bias [51] or population-level dependencies [52] are used to address these issues, resulting in high accuracy but biased errors [53]. AI algorithms can learn these biases from a single genome with a known gold standard of reference variant calls and produce superior variant calls. DeepVariant, a CNN-based variant caller trained directly on read alignments without any specialized knowledge about genomics or sequencing platforms, was recently shown to outperform standard tools on some variant-calling tasks [54]. The improved accuracy is thought to be due to the ability of CNNs to identify complex dependencies in sequencing data. In addition, recent results suggest that deep learning is poised to revolutionize base calling (and as a result, variant identification) for nanopore-based sequencing technologies, which have historically struggled to compete with established sequencing technology because of the error-prone nature of prior base-calling algorithms [55].

### Genome annotation and variant classification

After variant calling, the interpretation of human genome data relies on the identification of relevant genetic variants through prior knowledge and inference of the impact of genetic variants on functional genomic elements. AI algorithms can improve the use of prior knowledge by informing phenotype-to-genotype mapping (described in the next section). Here, we describe both genome annotation and variant classification because many of the AI algorithms that are used to predict the presence of a functional element from primary DNA sequence data are also used to predict the impact of a genetic variation on those functional elements.

#### Classification of coding variants

Many methods have been developed for the classification of nonsynonymous variants [56]. Some of these methods have been integrated into deep-learning-based meta-predictors (models that process and merge the predictions produced by several other predictors) that outperform both their individual predictive components and the combination of those predictive components when integrated using regression or other machine-learning approaches [57]. For example, the combined annotation-dependent depletion approach (CADD) [58] combines a variety of predictive features in a machine-learning algorithm to predict the deleteriousness of genetic variants. A deep-learning-based extension of CADD, named DANN, demonstrated improved performance using the same set of input features as CADD but combined in a deep neural network [57]. This technical extension of CADD suggests that deep learning may be a superior approach for integrating known features that are predictive of deleteriousness. However, the classification accuracies of these tools are not sufficient to drive clinical reporting, although they can be useful for guiding the interpretation of clinical genomic data by prioritizing potential candidate variants for further consideration.

More interesting are AI-based methods that make predictions directly from DNA or protein sequence data with minimal hand-crafting of features. One approach, PrimateAI, which used CNNs trained on variants of known pathogenicity with data augmentation using cross-species information, was shown to outperform prior methods when trained directly upon sequence alignments [59]. The network was able to learn important protein domains, conserved amino acid positions, and sequence dependencies directly from the training data consisting of about 120,000 human samples. PrimateAI substantially exceeded the performance of other variant pathogenicity prediction tools in differentiating benign and pathogenic de-novo mutations in candidate developmental disorder genes, and in reproducing prior knowledge in Clinvar [60]. These results suggest that PrimateAI is an important step forward for variant-classification tools that may lessen the reliance of clinical reporting on prior knowledge. In addition, deep generative models have shown promise for predicting the effects of genetic variants [61], and are especially intriguing given their ability to evaluate the joint influence of multiple genetic variants and/or complex indels on protein function, a capability that is largely absent from most pathogenicity prediction tools. Deep generative models are a type of deep neural network that can learn to replicate data distributions and produce examples not previously observed by the model. For example, a deep generative model trained on images of birds could learn to generate novel bird images.

#### Classification of non-coding variants

The computational identification and prediction of non-coding pathogenic variation is an open challenge in human genomics [62]. Recent findings suggest that AI algorithms will substantially improve our ability to understand non-coding genetic variation. Splicing defects in genes are responsible for at least 10% of rare pathogenic genetic variation [63], but they can be difficult to identify because of the complexity of intronic and exonic splicing enhancers, silencers, insulators, and other long range and combinatorial DNA interactions that influence gene splicing [64]. SpliceAI, a 32-layer deep neural network, is able to predict both canonical and non-canonical splicing directly from exon–intron junction sequence data [27]. Remarkably, SpliceAI was able to use long-range sequence information to boost prediction accuracy from 57%, using a short window size (80 nucleotides) typical for many prior splicing prediction tools, to 95% when a 10 kb window size was ingested by the AI algorithm, and was able to identify candidate cryptic splicing variants underlying neurodevelopmental disorders.

Deep-learning-based approaches have also substantially improved our ability to detect regulatory elements [65, 66] and to predict the influence of genetic variation on those elements. DeepSEA, a multitask hierarchically structured CNN trained on large-scale functional genomics data [67], was able to learn sequence dependencies at multiple scales and simultaneously produce predictions of DNase hypersensitive sites, transcription factor binding sites, histone marks, and the influence of genetic variation on those regulatory elements, with a level of accuracy superior to those of other tools for prioritizing non-coding functional variants [68]. As seen for SpliceAI, the ability of DeepSEA to ingest DNA sequences of 1 kb, which is substantially larger than the input to typical motif-based search tools, was critical to this improved performance. Extensions of DeepSEA have been applied to whole-genome sequencing data from families with autism spectrum disorder to reveal several candidate non-coding mutations [69]. Further extension to the ExPecto algorithm has demonstrated its ability to predict gene expression levels directly from DNA sequence information [70]. Further investigation of these new deep-learning based frameworks for the analysis of non-coding sequence data is likely to provide new insights into the regulatory code of the human genome.

### Phenotype-to-genotype mapping

Human genomes contain numerous genetic variants that are either previously described as pathogenic or predicted to be pathogenic [71], regardless of the individual health status [72]. Therefore, the molecular diagnosis of disease often requires both the identification of candidate pathogenic variants and a determination of the correspondence between the diseased individual’s phenotype and those expected to result from each candidate pathogenic variant. AI algorithms can significantly enhance the mapping of phenotype to genotype, especially through the extraction of higher-level diagnostic concepts that are embedded in medical images and EHRs.

#### Image to genetic diagnosis

The human phenotype ontology lists 1007 distinct terms defining different abnormalities of the face [73]. These abnormalities are associated with 4526 diseases and 2142 genes. A dysmorphologist will often identify these abnormalities individually and synthesize them into a clinical diagnosis. The clinical diagnosis may then inform targeted gene sequencing or phenotype-informed analysis of more comprehensive genetic data. Often the human-provided clinical diagnosis and molecular diagnoses overlap but do not match precisely because of the phenotypic similarity of genetically distinct syndromes. DeepGestalt, a CNN-based facial image analysis algorithm, dramatically outperforms human dysmorphologists in this task and is precise enough to distinguish between molecular diagnoses that are mapped to the same clinical diagnosis (that is, distinct molecular forms of Noonan syndrome) [19]. When combined with genomic data, PEDIA, a genome interpretation system incorporating DeepGestalt, was able to use phenotypic features extracted from facial photographs to accurately prioritize candidate pathogenic variants for 105 different monogenic disorders across 679 individuals [74]. Deployment of DeepGestalt as a face-scanning app has the potential to both democratize and revolutionize the identification of genetic syndromes [20].

Genetic syndromes that are identified through facial analysis can be readily confirmed with DNA testing, but adequate material for somatic mutation testing is not always available in some instances of cancer. Nevertheless, knowledge of the genomic underpinnings of a tumor are critical to treatment planning. Here again, AI can bridge the gap between image-derived phenotypes and their probable genetic source. A ‘survival CNN’, which is a combination of a CNN with Cox proportional hazards-based outcomes (a type of statistical survival analysis), was able to learn the histological features of brain tumors that are associated with survival and correlated with somatic mutation status [75]. Importantly, this algorithm was not trained to predict genomic aberrations directly. Inspection of the CNN concepts used to make the survival predictions identified novel histological features that are important for prognosis determination. Like the faces of individuals with phenotypically overlapping genetic syndromes, these results suggest that the genomic aberrations underpinning an individual’s tumor could potentially be predicted directly from tumor histology images. More generally, AI-based computer vision systems appear to be capable of predicting the genomic aberrations that are likely to be present in an individual’s genome on the basis of the complex phenotypes embedded in relevant clinical images [20, 75].

#### EHR to genetic diagnosis

Disease phenotypes can be complex and multimodal; captured not only by medical imaging, but also by biochemical and other tests that may be ordered at different times and perhaps by different physicians during the course of a differential diagnosis. These results are documented in an EHR where physicians synthesize these findings to provide diagnoses and inform clinical decision-making. Although human specialists can accomplish this task accurately within their area of expertise, AI-based algorithms can be general EHR pattern recognition experts. In a recent study involving more than 500,000 patients, an AI-based NLP approach was used to extract clinically relevant features from EHR data. A hierarchical statistical model, tiered on the basis of anatomic divisions in a manner meant to mimic the clinical reasoning of a composite of experienced physicians, was trained on the NLP output to generate a diagnostic system [48]. Overall, this system was able to differentiate between 55 common pediatric diagnoses with 92% accuracy.

When linked with genomic data, an AI-based diagnostic agent coupled with a genome interpretation system can rapidly produce genetic diagnoses. For example, an NLP system was designed to extract phenotypic descriptions automatically from EHR data of pediatric patients with rare diseases, and to rank matches to the expected phenotypic features of candidate pathogenic variants in the patients’ genomes [49]. In 101 children with 105 genetic diseases, automated retrospective genomic diagnoses agreed with expert human interpretation at 97% recall and 99% precision. The system was also able to provide automated genomic diagnoses prospectively for three of seven seriously ill ICU infants. Intriguingly, a simpler phenotypic risk score approach, applied to an adult population with EHR and genomic data, was able to identify previously unrecognized monogenic conditions in 18 individuals from a population of 21,701 [76]. These results suggest that AI-based phenotype-to-genotype mapping approaches could significantly improve the diagnostic yield of genetic testing and the identification of individuals with unrecognized genetic disorders.

### Genotype-to-phenotype prediction

Ultimately, the clinical purpose of genetics is to provide diagnoses and forecasts of future disease risk. Relatively simple statistical approaches to polygenic risk prediction allow for personally and clinically useful stratification of risk for some common complex diseases [77]. A few studies have attempted genomic prediction of complex human traits using AI algorithms, but most of those reported in the literature to date are probably overfit as they purportedly explain substantially more trait variance than should be possible on the basis of heritability estimates. One application of machine learning to genomic prediction of height was able to provide relatively accurate predictions within expected bounds [78], suggesting that AI-based methods can be used to improve upon statistical techniques. However, the true utility of AI-based approaches in genotype-to-phenotype prediction will probably come from the integration of a variety of health data types and risk factors into comprehensive predictors of disease risk.

Common diseases are a result of a complex interplay between inherited genetic risk factors, environmental exposures, and behaviors. Genetic risk alone provides a baseline estimate of lifetime risk for disease, but genetic risk combined with other risk factors allows for a narrowing of that probability space into a short-term projection of disease risk. For example, several non-genetic risk factors are associated with breast cancer risk, including mammographic density, age at first birth, age at menarche, and age at menopause. Combining these non-genetic risk factors with genetic data significantly improves the accuracy of breast cancer risk models and can inform risk-based mammographic screening strategies [79]. Similarly, significant improvement in risk stratification can be achieved by integrating conventional and genetic risk factors for coronary artery disease [80]. Genetic risk score models are more useful than simple pathogenicity assertions in cases where a common disease is the result of a combination of weak effects from multiple loci. However, current models integrate genetic and non-genetic risk factors in simple additive models that probably do not capture the complex causal relationships between these heterogenous risk factors. AI algorithms, given an appropriate volume of data, excel at dissecting this complexity. Unraveling the complex interplay between genetic data, EHR data, digital health monitoring devices, and other sources of health information with AI-based algorithms is a compelling prospect for the future.

## Challenges and limitations

AI-based algorithms can be superhuman in their ability to interpret complex data. However, their power and complexity can also result in spurious or even unethical and discriminatory conclusions when applied to human health data. Without careful consideration of the methods and biases embedded in a trained AI system, the practical utility of these systems in clinical diagnostics is limited. Thus, we end with a discussion on the challenges and limitations of AI in clinical diagnostics.

### Regulatory issues

A growing number of AI algorithms have been approved by the FDA [81]. These algorithms raise a number of regulatory and ethical challenges around the sourcing and privacy of the data used to train the algorithms [82], the transparency and generalizability of the underlying algorithms themselves, the regulatory process for refreshing these algorithms as further data become available, and the liability associated with prediction errors [83]. Some of these issues can and should be addressed by open sharing of AI models in detail (including source codes, model weights, meta graphs, and so on) with the scientific and medical community to improve transparency. Other issues will need to be addressed by the development of: (i) best practices for the interpretability of predictions to protect patient autonomy and shared decision-making; (ii) fairness standards to minimize disparities induced by machine bias; and (iii) ad hoc guidance to allow for continuous improvement of the algorithms [83]. As with most biomedical advances, the cost and expertise necessary to deploy AI algorithms is another concern, although these concerns diminish as interpretability and fairness issues are addressed. We explore these issues in further detail below.

### AI interpretability

AI is often criticized for being a ‘black box’: a system that produces an output without any explanation or justification. While this is perfectly acceptable in low-risk situations, clinical decision-making is not a low-risk situation. ‘What?’ may sufficiently encompass the question of interest in a general object-detection task, but ‘why?’ is an inherent part of the question in most clinical diagnostic tasks, because it is often crucial to subsequent clinical decision-making or at least necessary for acceptance of the prediction by both physicians and patients. An ideal AI-based clinical diagnostic system should produce accurate predictions and provide human-interpretable explanations of those predictions. A common approach to answering ‘why?’ in computer vision applications is to generate a visual overlay of the portions of an image that contribute most strongly to an output prediction [84, 85]. This strategy works well for image-based and other CNN-based clinical diagnostic tasks. In fact, many of the AI-based clinical diagnostic methods described in this review include some form of interpretive analysis. Thus, although AI interpretability is an important problem in general, the criticism of ‘black box’ systems in current AI-based clinical diagnostics may be overstated.

When complex interdependencies form the basis of a prediction, however, accurate interpretation of AI output becomes quite challenging [86]. Interpretable machine-learning methods are an active area of computer science research [87], but most interpretable AI approaches involve the production of a simplified and potentially inaccurate approximation of the more complex AI system [86]. Recently, a move towards more interactive models of interpretability through ‘dialogue’ with the AI system has been proposed [86]. This approach allows the human user to ask contrastive questions of the AI system in order to explore how its output predictions would change if inputs were modified. This approach could also facilitate a dialogue between physician and patient, with the aid of the AI interpretation system, to help them to understand the clinical diagnosis and, in some instances, the risk factors that could be modified to change the predicted outcome. Thus, further improvements to interpretable AI systems could not only substantially enhance the acceptability of AI predictions but also enhance the transparency of health communication between physicians and patients.

### Data and machine bias

Interpretative output is not only necessary for acceptance in clinical practice but is also important for unveiling the knowledge discovered by AI systems and for detecting biases that may result in undesirable behavior. There is substructure embedded in genomic and health data. Some substructure is due to truly differing causal relationships between alleged risk factors and health outcomes, whereas other substructure can be attributed to external factors such as socioeconomic status, cultural practices, unequal representation, and other non-causal factors that relate to the delivery and accessibility of medicine and clinical tests rather than to their efficacy [88, 89]. AI systems must be carefully applied to differentiate between these types of bias. When medical AI systems are not inspected for non-causal bias, they can act as propagators of disparity. For example, DeepGestalt, the previously described AI system for facial dysmorphology analysis, displayed poor accuracy for the identification of Down syndrome in individuals of African versus European ancestry (36.8% versus 80%, respectively) [90]. Retraining the model with examples of Down syndrome in individuals of African ancestry improved the diagnosis of Down syndrome in individuals of African ancestry to 94.7% [90]. Genetic risk prediction is also prone to unequal performance in different population groups because of underrepresentation in the training data [91].

However, not all machine bias can be resolved by addressing underrepresentation in training data. In some cases, the bias is embedded in ostensibly representative training data. For example, gender bias is common in written documents and can be rapidly incorporated into NLP systems [92]. Extensions to these models were required to ‘debias’ word embeddings. In clinical applications, EHR data may be representative overall, but the contents may include biases that result from the delivery of care or physician bias. For example, recent immigrants in Canada are more likely to receive aggressive care and die in intensive care units than are other residents [93]. Furthermore, the substructure of genomic data is correlated with population structure, which can lead to the appearance of non-causal trait associations [94]. However, tools that will help to address machine bias are being developed, and careful attention to these issues could not only help to resolve machine bias issues but could eventually lead to diagnostic systems that are free from human bias [95].

## Conclusions and future directions

AI systems have surpassed the performance of state-of-the-art methods and have gained FDA clearance for a variety of clinical diagnostics, especially imaging-based diagnostics. The availability of large datasets for training, for example, large collections of annotated medical images or large functional genomics datasets, in conjunction with advances in AI algorithms and in the GPU systems used to train them, is driving this surge of productivity. Currently, the most promising applications of AI in clinical genomics appear to be the AI extraction of deep phenotypic information from images, EHRs, and other medical devices to inform downstream genetic analysis. However, deep-learning algorithms have also shown tremendous promise in a variety of clinical genomics tasks such as variant calling, genome annotation, and functional impact prediction. It is possible that more generalized AI tools will become the standard in these areas, especially for clinical genomics tasks where inference from complex data (that is, variant calling) is a frequently recurring task. These applications have benefited from advances in CNNs and RNNs which appear to be particularly well suited for the analysis of genomic data. Yet, the utility of AI algorithms as the ultimate clinical decision support tool in predicting common complex human phenotypes has not been convincingly demonstrated. The rise of biobank-scale efforts with longitudinal health data collection, such as the UK Biobank [96] and All of Us Research Program [97], will potentially provide the training datasets necessary to make this goal a reality. Given the reliance of AI on large-scale training datasets, it is likely that the scalable collection of phenotype data, and not genomic data, will be the more difficult barrier to overcome in realizing this ambition. Modern DNA sequencing technology allows for the generation of genomic data uniformly and at scale, but the collection of phenotype data requires numerous data collection modes, and tends to be slow, expensive, and highly variable across collection sites. Finally, the interpretability and identification of machine bias are essential to broad acceptance of AI technology in any clinical diagnostic modality.

## Abbreviations

AI: Artificial intelligence
CADD: Combined annotation-dependent depletion approach
CNN: Convolutional neural network
EHR: Electronic health record
FDA: US Food and Drug Administration
GPU: Graphics processing unit
NLP: Natural language processing
RNN: Recurrent neural network

## References

[1] Torkamani A, Andersen KG, Steinhubl SR, Topol EJ (2017). High-definition medicine. Cell..

[2] Esteva A, Robicquet A, Ramsundar B, Kuleshov V, DePristo M, Chou K (2019). A guide to deep learning in healthcare. Nat Med.

[3] Fraser KC, Meltzer JA, Rudzicz F (2016). Linguistic features identify Alzheimer’s disease in narrative speech. J Alzheimers Dis.

[4] Rajkomar A, Oren E, Chen K, Dai AM, Hajaj N, Liu PJ (2018). Scalable and accurate deep learning for electronic health records. NPJ Digit Med.

[5] Zou J, Huss M, Abid A, Mohammadi P, Torkamani A, Telenti A (2019). A primer on deep learning in genomics. Nat Genet.

[6] Eraslan G, Avsec Ž, Gagneur J, Theis FJ (2019). Deep learning: new computational modelling techniques for genomics. Nat Rev Genet..

[7] Retson TA, Besser AH, Sall S, Golden D, Hsiao A (2019). Machine learning and deep neural networks in thoracic and cardiovascular imaging. J Thorac Imaging.

[8] Asch FM, Abraham T, Jankowski M, Cleve J, Adams M, Romano N (2019). Accuracy and reproducibility of a novel artificial intelligence deep learning-based algorithm for automated calculation of ejection fraction in echocardiography. J Am Coll Cardiol.

[9] Le EPV, Wang Y, Huang Y, Hickman S, Gilbert FJ (2019). Artificial intelligence in breast imaging. Clin Radiol.

[10] Majumdar A, Brattain L, Telfer B, Farris C, Scalera J (2018). Detecting intracranial hemorrhage with deep learning. Conf Proc IEEE Eng Med Biol Soc.

[11] FDA approves stroke-detecting AI software. Nat Biotechnol. 2018;36:290. 10.1038/nbt0418-290. 10.1038/nbt0418-290 29621226

[12] Gulshan V, Peng L, Coram M, Stumpe MC, Wu D, Narayanaswamy A (2016). Development and validation of a deep learning algorithm for detection of diabetic retinopathy in retinal fundus photographs. JAMA..

[13] van der Heijden AA, Abramoff MD, Verbraak F, van Hecke MV, Liem A, Nijpels G (2018). Validation of automated screening for referable diabetic retinopathy with the IDx-DR device in the Hoorn diabetes care system. Acta Ophthalmol.

[14] Evans AJ, Bauer TW, Bui MM, Cornish TC, Duncan H, Glassy EF (2018). US Food and Drug Administration approval of whole slide imaging for primary diagnosis: a key milestone is reached and new questions are raised. Arch Pathol Lab Med.

[15] Esteva A, Kuprel B, Novoa RA, Ko J, Swetter SM, Blau HM (2017). Dermatologist-level classification of skin cancer with deep neural networks. Nature..

[16] Niazi MKK, Parwani AV, Gurcan MN (2019). Digital pathology and artificial intelligence. Lancet Oncol.

[17] Rios Velazquez E, Parmar C, Liu Y, Coroller TP, Cruz G, Stringfield O (2017). Somatic mutations drive distinct imaging phenotypes in lung cancer. Cancer Res.

[18] Coudray N, Ocampo PS, Sakellaropoulos T, Narula N, Snuderl M, Fenyö D (2018). Classification and mutation prediction from non-small cell lung cancer histopathology images using deep learning. Nat Med.

[19] Gurovich Y, Hanani Y, Bar O, Nadav G, Fleischer N, Gelbman D (2019). Identifying facial phenotypes of genetic disorders using deep learning. Nat Med.

[20] Dolgin E. AI face-scanning app spots signs of rare genetic disorders. Nature. 2019. 10.1038/d41586-019-00027-x.

[21] Poplin R, Varadarajan AV, Blumer K, Liu Y, McConnell MV, Corrado GS (2018). Prediction of cardiovascular risk factors from retinal fundus photographs via deep learning. Nat Biomed Eng.

[22] Hannun AY, Rajpurkar P, Haghpanahi M, Tison GH, Bourn C, Turakhia MP (2019). Cardiologist-level arrhythmia detection and classification in ambulatory electrocardiograms using a deep neural network. Nat Med.

[23] Tison GH, Sanchez JM, Ballinger B, Singh A, Olgin JE, Pletcher MJ (2018). Passive detection of atrial fibrillation using a commercially available smartwatch. JAMA Cardiol.

[24] Attia ZI, Kapa S, Lopez-Jimenez F, McKie PM, Ladewig DJ, Satam G (2019). Screening for cardiac contractile dysfunction using an artificial intelligence-enabled electrocardiogram. Nat Med.

[25] Galloway CD, Valys AV, Shreibati JB, Treiman DL, Petterson FL, Gundotra VP (2019). Development and validation of a deep-learning model to screen for hyperkalemia from the electrocardiogram. JAMA Cardiol.

[26] Leung MKK, Xiong HY, Lee LJ, Frey BJ (2014). Deep learning of the tissue-regulated splicing code. Bioinformatics..

[27] Jaganathan K, Kyriazopoulou Panagiotopoulou S, McRae JF, Darbandi SF, Knowles D, Li YI (2019). Predicting splicing from primary sequence with deep learning. Cell.

[28] Quang D, Xie X (2016). DanQ: a hybrid convolutional and recurrent deep neural network for quantifying the function of DNA sequences. Nucleic Acids Res.

[29] Wang J, Cao H, Zhang JZH, Qi Y (2018). Computational protein design with deep learning neural networks. Sci Rep.

[30] Li J, Deng L, Haeb-Umbach R, Gong Y, Li J, Deng L, Li J, Deng L, Haeb-Umbach R, Gong Y (2016). Fundamentals of speech recognition. Robust automatic speech recognition: a bridge to practical applications.

[31] Parthasarathy S, Rozgic V, Sun M, Wang C. Improving emotion classification through variational inference of latent variables. In: International Conference on Acoustics, Speech and Signal Processing (ICASSP)—Proceedings. IEEE. 2019:7410–4 https://ieeexplore.ieee.org/document/8682823. Accessed 31 Oct 2019.

[32] Trigeorgis G, Ringeval F, Brueckner R, Marchi E, Nicolaou MA, Schuller B, et al. Adieu features? End-to-end speech emotion recognition using a deep convolutional recurrent network. In: International Conference on Acoustics, Speech and Signal Processing (ICASSP)—Proceedings. IEEE. 2016:5200–4 https://ieeexplore.ieee.org/document/7472669. Accessed 31 Oct 2019.

[33] Hinton G, Deng L, Yu D, Dahl GE, Mohamed A, Jaitly N (2012). Deep neural networks for acoustic modeling in speech recognition. IEEE Signal Process Mag.

[34] Prabhavalkar R, Rao K, Sainath TN, Li B, Johnson L, Jaitly N (2017). A Comparison of sequence-to-sequence models for speech recognition. In: Proceedings of the Annual Conference of the International Speech Communication Association, Interspeech.

[35] Li Zhichao, Huang Jilin, Hu Zhiping (2019). Screening and Diagnosis of Chronic Pharyngitis Based on Deep Learning. International Journal of Environmental Research and Public Health.

[36] Zhan A, Mohan S, Tarolli C, Schneider RB, Adams JL, Sharma S (2018). Using smartphones and machine learning to quantify Parkinson disease severity the mobile Parkinson disease score. JAMA Neurol.

[37] Ringeval F, Schuller B, Valstar M, Ni C, Cowie R, Tavabi L, et al. AVEC 2019 workshop and challenge: state-of-mind, detecting depression with AI, and cross-cultural affect recognition. In: Proceedings of the 9th International on Audio/Visual Emotion Challenge and Workshop. Nice; 2019. p. 3–12. 10.1145/3347320.3357688.

[38] Marmar CR, Brown AD, Qian M, Laska E, Siegel C, Li M (2019). Speech-based markers for posttraumatic stress disorder in US veterans. Depress Anxiety.

[39] Maor E, Sara JD, Orbelo DM, Lerman LO, Levanon Y, Lerman A (2018). Voice signal characteristics are independently associated with coronary artery disease. Mayo Clin Proc.

[40] Mohr DN, Turner DW, Pond GR, Kamath JS, De Vos CB, Carpenter PC (2003). Speech recognition as a transcription aid: a randomized comparison with standard transcription. J Am Med Informatics Assoc.

[41] Edwards Erik, Salloum Wael, Finley Greg P., Fone James, Cardiff Greg, Miller Mark, Suendermann-Oeft David (2017). Medical Speech Recognition: Reaching Parity with Humans. Speech and Computer.

[42] Wu Y, Schuster M, Chen Z, Le QV, Norouzi M, Macherey W (2016). Google’s neural machine translation system: bridging the gap between human and machine translation. arXiv.

[43] Collobert R, Weston J. A unified architecture for natural language processing: deep neural networks with multitask learning. In: ICML '08. Proceedings of the 25th International Conference on Machine learning. Helsinki; 2008, 2008. p. 160–7. 10.1145/1390156.1390177.

[44] Miotto R, Li L, Kidd BA, Dudley JT (2016). Deep patient: an unsupervised representation to predict the future of patients from the electronic health records. Sci Rep.

[45] Chen J, Druhl E, Polepalli Ramesh B, Houston TK, Brandt CA, Zulman DM (2018). A natural language processing system that links medical terms in electronic health record notes to lay definitions: system development using physician reviews. J Med Internet Res.

[46] Kohut K, Limb S, Crawford G (2019). The changing role of the genetic counsellor in the genomics era. Curr Genet Med Rep.

[47] Diller G-P, Kempny A, Babu-Narayan SV, Henrichs M, Brida M, Uebing A (2019). Machine learning algorithms estimating prognosis and guiding therapy in adult congenital heart disease: data from a single tertiary Centre including 10,019 patients. Eur Heart J.

[48] Liang H, Tsui BY, Ni H, Valentim CCS, Baxter SL, Liu G (2019). Evaluation and accurate diagnoses of pediatric diseases using artificial intelligence. Nat Med.

[49] Clark MM, Hildreth A, Batalov S, Ding Y, Chowdhury S, Watkins K (2019). Diagnosis of genetic diseases in seriously ill children by rapid whole-genome sequencing and automated phenotyping and interpretation. Sci Transl Med.

[50] Li H (2014). Toward better understanding of artifacts in variant calling from high-coverage samples. Bioinformatics..

[51] DePristo MA, Banks E, Poplin R, Garimella KV, Maguire JR, Hartl C (2011). A framework for variation discovery and genotyping using next-generation DNA sequencing data. Nat Genet.

[52] Garrison E, Marth G (2012). Haplotype-based variant detection from short-read sequencing. arXiv.

[53] Hwang S, Kim E, Lee I, Marcotte EM (2015). Systematic comparison of variant calling pipelines using gold standard personal exome variants. Sci Rep.

[54] Poplin R, Chang PC, Alexander D, Schwartz S, Colthurst T, Ku A (2018). A universal SNP and small-indel variant caller using deep neural networks. Nat Biotechnol.

[55] Wick RR, Judd LM, Holt KE (2019). Performance of neural network basecalling tools for Oxford nanopore sequencing. Genome Biol.

[56] Tang H, Thomas PD (2016). Tools for predicting the functional impact of nonsynonymous genetic variation. Genetics..

[57] Quang D, Chen Y, Xie X (2015). DANN: a deep learning approach for annotating the pathogenicity of genetic variants. Bioinformatics..

[58] Kircher M, Witten DM, Jain P, O’Roak BJ, Cooper GM, Shendure J (2014). A general framework for estimating the relative pathogenicity of human genetic variants. Nat Genet.

[59] Sundaram L, Gao H, Padigepati SR, McRae JF, Li Y, Kosmicki JA (2018). Predicting the clinical impact of human mutation with deep neural networks. Nat Genet.

[60] Landrum MJ, Lee JM, Benson M, Brown GR, Chao C, Chitipiralla S (2018). ClinVar: improving access to variant interpretations and supporting evidence. Nucleic Acids Res.

[61] Riesselman AJ, Ingraham JB, Marks DS (2018). Deep generative models of genetic variation capture the effects of mutations. Nat Methods.

[62] Chatterjee S, Ahituv N (2017). Gene regulatory elements, major drivers of human disease. Annu Rev Genomics Hum Genet.

[63] Soemedi R, Cygan KJ, Rhine CL, Wang J, Bulacan C, Yang J (2017). Pathogenic variants that alter protein code often disrupt splicing. Nat Genet.

[64] Baeza-Centurion P, Miñana B, Schmiedel JM, Valcárcel J, Lehner B (2019). Combinatorial genetics reveals a scaling law for the effects of mutations on splicing. Cell..

[65] Kelley DR, Reshef YA, Bileschi M, Belanger D, McLean CY, Snoek J (2018). Sequential regulatory activity prediction across chromosomes with convolutional neural networks. Genome Res.

[66] Alipanahi B, Delong A, Weirauch MT, Frey BJ (2015). Predicting the sequence specificities of DNA- and RNA-binding proteins by deep learning. Nat Biotechnol.

[67] Bernstein BE, Stamatoyannopoulos JA, Costello JF, Ren B, Milosavljevic A, Meissner A (2010). The NIH roadmap epigenomics mapping consortium. Nat Biotechnol.

[68] Zhou J, Troyanskaya OG (2015). Predicting effects of noncoding variants with deep learning-based sequence model. Nat Methods.

[69] Zhou J, Park CY, Theesfeld CL, Wong AK, Yuan Y, Scheckel C (2019). Whole-genome deep-learning analysis identifies contribution of noncoding mutations to autism risk. Nat Genet.

[70] Zhou J, Theesfeld CL, Yao K, Chen KM, Wong AK, Troyanskaya OG (2018). Deep learning sequence-based ab initio prediction of variant effects on expression and disease risk. Nat Genet.

[71] Telenti A, Pierce LCT, Biggs WH, Di Iulio J, Wong EHM, Fabani MM (2016). Deep sequencing of 10,000 human genomes. Proc Natl Acad Sci U S A.

[72] Erikson GA, Bodian DL, Rueda M, Molparia B, Scott ER, Scott-Van Zeeland AA (2016). Whole-genome sequencing of a healthy aging cohort. Cell..

[73] Köhler S, Carmody L, Vasilevsky N, Jacobsen JOB, Danis D, Gourdine JP (2019). Expansion of the human phenotype ontology (HPO) knowledge base and resources. Nucleic Acids Res.

[74] Hsieh T-C, Mensah MA, Pantel JT, Aguilar D, Bar O, Bayat A, et al. PEDIA: prioritization of exome data by image analysis. Genet Med. 2019. 10.1038/s41436-019-0566-2. 10.1038/s41436-019-0566-2 PMC6892739 31164752

[75] Mobadersany P, Yousefi S, Amgad M, Gutman DA, Barnholtz-Sloan JS, Velázquez Vega JE (2018). Predicting cancer outcomes from histology and genomics using convolutional networks. Proc Natl Acad Sci U S A.

[76] Bastarache L, Hughey JJ, Hebbring S, Marlo J, Zhao W, Ho WT (2018). Phenotype risk scores identify patients with unrecognized mendelian disease patterns. Science..

[77] Torkamani A, Wineinger NE, Topol EJ (2018). The personal and clinical utility of polygenic risk scores. Nat Rev Genet.

[78] Lello L, Avery SG, Tellier L, Vazquez AI (2018). de los Campos G, Hsu SDH. Accurate genomic prediction of human height. Genetics.

[79] Lee A, Mavaddat N, Wilcox AN, Cunningham AP, Carver T, Hartley S (2019). BOADICEA: a comprehensive breast cancer risk prediction model incorporating genetic and nongenetic risk factors. Genet Med..

[80] Inouye M, Abraham G, Nelson CP, Wood AM, Sweeting MJ, Dudbridge F (2018). Genomic risk prediction of coronary artery disease in 480,000 adults. J Am Coll Cardiol.

[81] Topol EJ (2019). High-performance medicine: the convergence of human and artificial intelligence. Nat Med.

[82] Lomas N. Google has used contract swaps to get bulk access terms to NHS patient data. TechCrunch. 2019; https://techcrunch.com/2019/10/22/google-has-used-contract-swaps-to-get-bulk-access-terms-to-nhs-patient-data/. Accessed 31 Oct 2019.

[83] Vayena E, Blasimme A, Cohen IG (2018). Machine learning in medicine: addressing ethical challenges. PLoS Med.

[84] Selvaraju RR, Cogswell M, Das A, Vedantam R, Parikh D, Batra D. Grad-CAM: visual explanations from deep networks via gradient-based localization. In: International Conference on Computer Vision (ICCV): IEEE; 2017. p. 618–26. http://ieeexplore.ieee.org/document/8237336/ Accessed 12 Aug 2019.

[85] Olah C, Mordvintsev A, Schubert L (2017). Feature visualization: how neural networks build up their understanding of images. Distill..

[86] Mittelstadt B, Russell C, Wachter S. Explaining explanations in AI. In: FAT* 2019. Proceedings of the 2019 Conference on Fairness, Accountability, and Transparency. Atlanta; 2019. p. 29, 279–31, 288. 10.1145/3287560.3287574.

[87] Doshi-Velez F, Kim B (2017). Towards a rigorous science of interpretable machine learning. arXiv.

[88] Gianfrancesco MA, Tamang S, Yazdany J, Schmajuk G (2018). Potential biases in machine learning algorithms using electronic health record data. JAMA Intern Med.

[89] Sirugo G, Williams SM, Tishkoff SA (2019). The missing diversity in human genetic studies. Cell.

[90] Lumaka A, Cosemans N, Lulebo Mampasi A, Mubungu G, Mvuama N, Lubala T (2017). Facial dysmorphism is influenced by ethnic background of the patient and of the evaluator. Clin Genet.

[91] Martin AR, Kanai M, Kamatani Y, Okada Y, Neale BM, Daly MJ (2019). Clinical use of current polygenic risk scores may exacerbate health disparities. Nat Genet.

[92] Bolukbasi T, Chang K-W, Zou JY, Saligrama V, Kalai AT. Man is to computer programmer as woman is to homemaker? Debiasing word embeddings. In: Lee DD, Sugiyama M, Luxburg UV, Guyon I, Garnett R, editors. Advances in neural information processing systems 29. Proceedings of the 30th Conference on Neural Information Processing Systems (NIPS 2016). Barcelona. p. 4349–57. https://papers.nips.cc/paper/6228-man-is-to-computer-programmer-as-woman-is-to-homemaker-debiasing-word-embeddings.pdf Accessed 31 Oct 2019.

[93] Yarnell CJ, Fu L, Manuel D, Tanuseputro P, Stukel T, Pinto R (2017). Association between immigrant status and end-of-life care in Ontario, Canada. JAMA.

[94] Sohail M, Maier RM, Ganna A, Bloemendal A, Martin AR, Turchin MC, et al. Polygenic adaptation on height is overestimated due to uncorrected stratification in genome-wide association studies. Elife. 2019;8. 10.7554/eLife.39702. 10.7554/eLife.39702 PMC6428571 30895926

[95] Chen IY, Szolovits P, Ghassemi M (2019). Can AI help reduce disparities in general medical and mental health care?. AMA J Ethics.

[96] Sudlow C, Gallacher J, Allen N, Beral V, Burton P, Danesh J (2015). UK biobank: an open access resource for identifying the causes of a wide range of complex diseases of middle and old age. PLoS Med.

[97] Sankar PL, Parker LS (2017). The precision medicine initiative’s all of us research program: an agenda for research on its ethical, legal, and social issues. Genet Med.

